# Pan-Asian Resuscitation Outcomes Study (PAROS): A Living Population Laboratory Transforming Resuscitation Science Across Asia

**DOI:** 10.1016/j.jacasi.2025.09.016

**Published:** 2025-12-02

**Authors:** Andrew Fu Wah Ho, Sang Do Shin, Marcus Eng Hock Ong

**Affiliations:** a Department of Emergency Medicine, Singapore General Hospital, Singapore; b Centre for Population Health Research and Implementation, SingHealth Regional Health System, Singapore; c Laboratory of Emergency Medical Services, Seoul National University Hospital Biomedical Research Institute, Seoul, Republic of Korea; d Department of Emergency Medicine, Seoul National University College of Medicine and Hospital, Seoul, Republic of Korea; e Health Services Research & Population Health, Duke-NUS Medical School, Singapore

**Keywords:** bystander cardiopulmonary resuscitation, cardiac arrest, emergency medical services, out-of-hospital cardiac arrest, resuscitation

**Figure:**
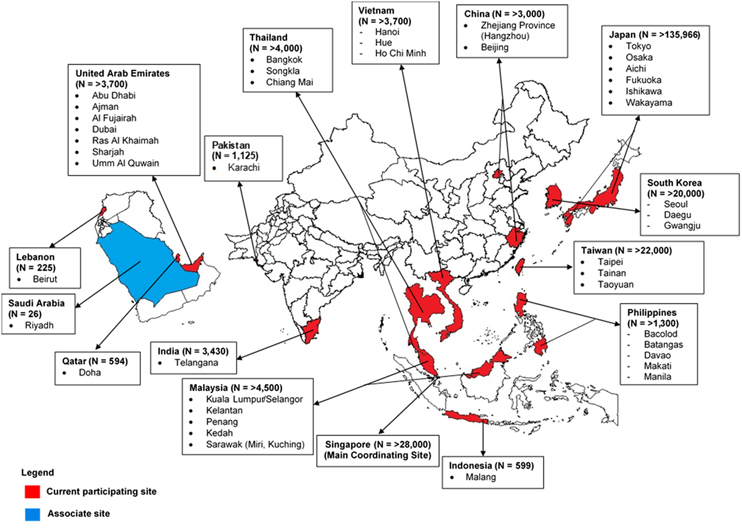

Out-of-hospital cardiac arrest (OHCA) is the loss of functional cardiac mechanical activity in association with an absence of systemic circulation occurring outside of a hospital setting. As a leading cause of mortality, it poses a major public health challenge worldwide.^1^ Asia, home to a majority of the global population, bears a disproportionate share of the global OHCA burden. Prior studies have found Asia to have the highest weighted incidence of OHCA and yet the lowest survival.^2^ It is also in Asia where large variations in OHCA epidemiology exist, contributed to by heterogeneity in population characteristics, emergency health systems characteristics, and resuscitation practices. Despite improving emergency medical services (EMS) and increasing public health investments in many parts of Asia, survival from OHCA remains poor and highly variable across the region.

Historically, efforts to understand and improve OHCA outcomes in Asia have been limited by absent or fragmented data and differences in reporting. Further, as outcomes after OHCA hinge heavily on an optimized chain of survival, crucial contextual data on community, EMS care processes, and resuscitation practices were needed.^3^ As an example, Asian EMS systems span the spectrum of well-established and robust systems (eg, South Korea), whereas others are still building basic prehospital response capacity. An understanding of these disparities in baseline and contextual situations is needed to design and implement strategies to improve OHCA outcomes in Asia.^4^

The PAROS (Pan-Asian Resuscitation Outcomes Study) study was established in 2010 as a response to this critical knowledge gap, aiming to build a collaborative OHCA registry to drive data-driven improvements in cardiac arrest care across Asia.^5^ For more than a decade, PAROS has provided standardized, population-level data on OHCA to track incidence, treatment and outcomes. Initially, these insights enabled benchmarking, understanding of gaps (eg, low bystander cardiopulmonary rates), and design of targeted interventions. Later, PAROS served as a platform for multinational trials of health system interventions. More recently, PAROS has informed global resuscitation strategies in low- to middle-income countries and has spearheaded efforts for a global coalition of resuscitation registries among other achievements. To date, PAROS has involved more than 40 sites in 15 countries, capturing more than 200,000 cases. In this paper we discuss the PAROS journey in driving improvements in OHCA care across Asia, highlighting pertinent aspects of its historical development, methodology, impact, challenges, and future directions.

## The registry and its evolution

PAROS is a prospective, long-term, multicenter, international OHCA registry involving EMS systems, hospitals, and academic collaborators across the Asia-Pacific. Conceptually, it operates on a low-cost, self-funded model of a collaborative research network, which is crucial to engage the spectrum of sites in Asia. At the time, existing OHCA registries, namely, CARES (Cardiac Arrest Registry to Enhance Survival, from the United States), the Canadian OPALS (Ontario Prehospital Advanced Life Support), and the ROC (Resuscitation Outcomes Consortium, from the United States) all originated from high-income countries with well-developed EMS systems. There had been a gap in understanding how systems with fewer resources can learn meaningfully from these registries and adopt best practices that are relevant to them.

**Figure 1:**
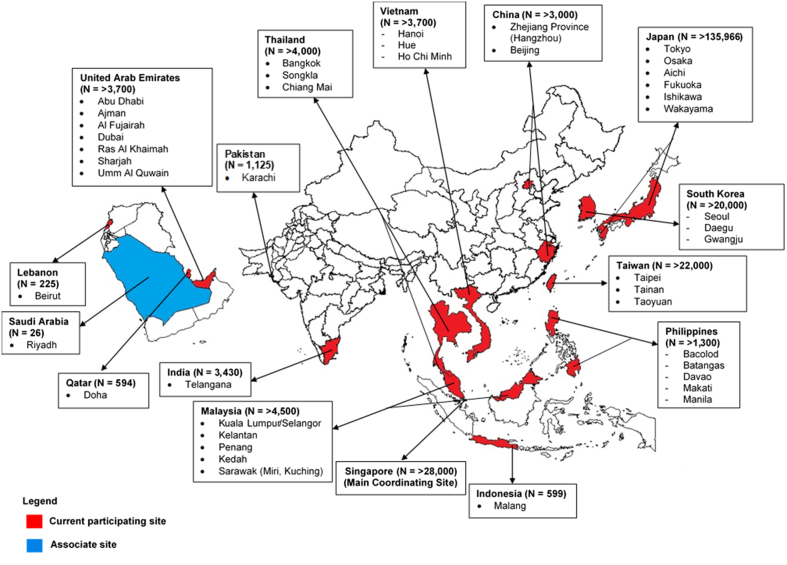
Map of PAROS CRN Current study sites and estimated data contribution in the PAROS (Pan-Asian Resuscitation Outcomes Study) clinical research network since established in 2010.

Participating sites were initially gathered through the Asian Association for Emergency Medical Services (then known as Asian EMS Council) which was formed around the same time. In 2010, the PAROS clinical research network (CRN) was formed. In the same year, PAROS formed an executive committee and adopted a formal constitution. The CRN is a nonprofit, investigator-led group committed to improving resuscitation outcomes through collaborative research. More information about the network can be found at the PAROS CRN website.^6^ A map of the PAROS CRN with data contribution is shown in Figure 1.

Over the years, PAROS has evolved from a descriptive registry into a platform supporting epidemiological and health systems research, system benchmarking, quality improvement, policy evaluation, and clinical trials.

## Design

The case definition is any OHCA conveyed by EMS or presenting at emergency departments as confirmed by the absence of pulse, unresponsiveness, and apnea. This includes both presumed cardiac and noncardiac etiologies. Patients are excluded if resuscitation was not attempted, such as those with decapitation, rigor mortis, dependent lividity, and known “do not attempt resuscitation” orders.

Each participating country is responsible for administrating its own data collection process. Data are input via a secured online electronic data capture system hosted by the study coordination center (SCC) in Singapore. Countries that already had national registries (ie, Singapore, South Korea, Japan, and Taiwan) could batch export data to the SCC.

To standardize data structures between sites and allow comparison with global data, PAROS’ unified data dictionary and taxonomy was developed in collaboration with CARES, and definitions follow the Utstein style. Data are collected from EMS dispatch records, ambulance records, hospital records, and death certificates. Data elements include patient demographics, arrest characteristics, EMS response, interventions, and survival outcomes. Non-core elements (not collected by all sites) include health-related quality of life. The primary outcome is survival to hospital discharge and survival to 30 days. Collected data are verified by local coordinators and then again by the SCC. Each participating country obtained and maintained intuitional review board approvals. A register of all institutional review board approvals is maintained at the SCC.

The PAROS data agreement developed in 2010 sets out the framework for data use within the PAROS CRN. Membership requires contributing a core dataset from EMS and/or hospital sources, with additional data encouraged where available. All contributed data are de-identified and belong to the network, with the SCC serving as custodian. Members retain ownership of their own source data but require approval for collaborative use of network data. Data requests must be submitted as research proposals, reviewed by 1 of 4 publications committees (OHCA, EMS Systems, emergency department, and EMS Survey), with approvals based on scientific merit and feasibility. Members must acknowledge PAROS CRN in their publications. Use and disclosure of data are restricted to approved purposes, with obligations to maintain confidentiality and security.

## Impact and contributions highlights

Since its inception, PAROS has made various contributions to both local and global resuscitation science. Capacity building is a key legacy of PAROS. Through collaborative publications, mentorship, and shared protocols, the network has strengthened research capabilities across Asia. PAROS has also facilitated South-South learning, where more developed EMS systems support emerging ones through training and data sharing. Findings from the registry have created knowledge, informed national EMS reforms, public health programs, and policy decisions in member countries. The CRN has published more than 100 peer-reviewed studies, serving as a model for regional registry collaboration in Asia. Some contributions have included: 1) the first multinational OHCA registry in Asia, allowing data from diverse systems to coalesce into a single collaborative registry^5^; 2) characterized OHCA epidemiology in Asia,^7^ and in doing so, revealed wide disparities in processes and outcomes, and described these in relation to health systems and EMS characteristics^8^, ^9^, ^10^, ^11^ — this also includes the first ever reports of OHCA data from several member countries; 3) identified key system gaps and levers for improvement, informing the design of trials, programs, and policies centered on improving basic life support, and in particular, bystander response^12^; 4) showed through a pragmatic multinational trial that telephone cardiopulmonary resuscitation (CPR) implementation in developing EMS systems is feasible and effective in improving bystander CPR rates and OHCA survival^13^^,^^14^; 5) shaped national policies, clinical guidelines (such as the International Liaison Committee on Resuscitation evidence evaluation process and the Utstein-style recommendations), EMS protocols and resuscitative practices in member countries and beyond such as on prehospital health system strategies,^15^ good Samaritan laws, CPR training programs,^16^ ambulance deployment and response times,^17^ public access defibrillator programs and prehospital airway management,^18^ pediatric resuscitation,^19^ telephone CPR,^20^ and termination of resuscitation protocols^21^^,^^22^ —these contributions in shaping international consensus statements and guidelines help ensure that global resuscitation recommendations are informed by Asian data; 6) it is a forerunner in the adoption of a long-term time horizon in assessing outcomes of OHCA survivors, following up patients beyond the first year of survival^23^^,^^24^; 7) contributed to global understanding of disparities in OHCA care and outcomes, such as on account of gender, aging,^25^ socioeconomic position, regional income,^26^ and location^27^^,^^28^; 8) Served as a “living laboratory” for testing interventions, such as community responder systems, public CPR training programs, mobile health technologies, and predictive analytics; 9) enabled important epidemiological studies in other fields such as on the health effects of air pollution,^29^ stroke, and acute myocardial infarction epidemiology; 10) the CRN serves a platform to both inform best practices in improving OHCA (eg, the Global Resuscitation Alliance’s 10 Steps Program), as well as implement them among member states; 11) the CRN serves as a platform to incubate registries for other time-sensitive medical emergencies such as trauma (Pan-Asia Trauma Outcome Study)^30^; 12) sounded the alarm on and explained the adverse impact of the COVID-19 pandemic on OHCA epidemiology and care processes^31^^,^^32^; 13) was one of the founding members of the Global OHCA registry (GOHCAR) consortium of registries, which drives efforts to increase value from OHCA registries through sharing best practices and innovative approaches of data sharing^33^; and 14) has served as a platform to enable early career researchers in resuscitation science.

## Challenges and future directions

PAROS aims to be a long-term registry, continuing to generate relevant insights to improve OHCA outcomes in diverse communities. There has been a need to continually identify challenges and address them to remain relevant and sustainable. Some of these include:

### Translation of knowledge into policy

Despite robust data, policy change can be slow and depends on political will, public awareness, and system readiness. Stronger knowledge translation strategies to influence policy consistently across regions must be developed. PAROS is leading efforts in implementation science to overcome the implementation gap.

### Funding disparities

Funding disparities also hinder equitable and sustained participation. Although some member sites benefit from robust institutional support, others face barriers in sustaining registry contributions. As representativeness is an important aspect of the usefulness of the registry, there is a need to continue to engage existing and potential sites.

### Sustainability of engagement

Continued engagement of sites depends on local leadership, institutional support, and perceived value. Continuous motivation through outreach, capacity building, recognition, and clear demonstration of impact is essential to maintain long-term participation.

### Inconsistent data quality and completeness

Not all participating countries have the same resources for data collection, entry, and validation. Data fragmentation between EMS and hospital systems limits the ability to follow the patient journey from arrest through to post-discharge outcomes. Many countries lack integrated health information systems, making it difficult to link prehospital and in-hospital records. This hinders comprehensive outcome assessment and impairs research into the full chain of survival. Member sites must continually identify and leverage opportunities to build high quality data pipelines such as linkage with hospital data systems.

### Data privacy

The increasing data privacy burden in some jurisdictions has introduced regulatory complexities. Compliance with local data protection laws, ethical review requirements, and cross-border data sharing policies varies widely. These evolving requirements demand robust governance frameworks that need continual development to enable unimpeded collaborative analysis. This may involve the use of privacy-preserving technologies such as federated data systems.

## Conclusions

Addressing several challenges noted above, there are now growing opportunities for data linkage with various health data sources. For instance, linkage with individual health records such as national health insurance systems can serve as a platform for longitudinal studies on outcomes such as disability and return to work among OHCA survivors. Furthermore, integrating with personal health screening records can form the basis for cohort studies tracking the occurrence of OHCA. In the future, it may be used as foundational data for personalized risk prediction of OHCA in line with technological advancements.

Ultimately, the success of PAROS will depend on sustained collaboration, mutual trust, and a shared vision to reduce preventable deaths and disability from OHCA in Asia. PAROS is deeply invested in the strengthening of health systems across Asia, with the aim that no one should experience a preventable death from OHCA due to lack of access to high-quality resuscitation care — whether in the community, through EMS, or in hospitals.

## Funding Support and Author Disclosures

This study has been supported by grants from the National Medical Research Council, Singapore, under the Clinician Scientist Award, Singapore (NMRC/CSA/024/2010, NMRC/CSA/0049/2013 and NMRC/CSA-SI/0014/2017), Ministry of Health, Health Services Research Grant, Singapore (HSRG/0021/2012), and the Singapore Translational Research Investigator Award (MOH-000982-01). Dr Ho has received grants from the SingHealth Duke-NUS Academic Medical Centre under the Clinician Investigator Advancement Programme (award number 15/FY2022/CIVA/03-A03) and National Medical Research Council (NMRC/CS_Seedfd/012/2018); and he has received conference support from Zoll Medical. Dr Ong has received funding from the Zoll Medical Corporation for a study involving mechanical cardiopulmonary resuscitation devices; has received grants from the Laerdal Foundation (20040), Laerdal Medical, and Ramsey Social Justice Foundation for funding of the Pan-Asian Resuscitation Outcomes Study; has had an advisory relationship with Global Healthcare SG, a commercial entity that manufactures cooling devices; and has received funding from Laerdal Medical on an observation program to their Community CPR Training Centre Research Program in Norway. Dr Shin has reported that they have no relationships relevant to the contents of this paper to disclose.
