# Supplementary material for: Pan-Asian Resuscitation Outcomes Study (PAROS): A Living Population Laboratory Transforming Resuscitation Science Across Asia
Source: JACC Asia. 2025 Dec 2;5(12):1642–7. doi: 10.1016/j.jacasi.2025.09.016 (PMC12802351; doi:10.1016/j.jacasi.2025.09.016)
Supplement: Supplemental Document [file mmc1.docx]

**Pan-Asian Resuscitation Outcomes Study Clinical Research Network**

**Country and Site Investigators**

**Japan**

Hideharu Tanaka (Kokushikan University, Tokyo); Kentaro Kajino (Kansai Medical University, Osaka); Koshi Nakagawa (Kokushikan University, Tokyo); Takashi Tagami (Nippon Medical School Tama Nagayama Hospital, Tokyo); Tatsuya Nishiuchi (Graduate School of Medical Sciences and Faculty of Medicine, Kindai University, Osaka); Takashi Nakagawa (Aichi Medical University Hospital; Aichi); Takahiro Hara (Osaka University, Osaka); Hiroshi Takyu (Kokushikan University, Tokyo); Yohei Okada (Kyoto University, Kyoto)

**South Korea**

Hyun Wook Ryoo (Kyungpook National University, Daegu); Hyun Ho Ryu (Chonnam National University Medical School and Hospital, Gwangju); Ki Jeong Hong (Seoul National University Hospital, Seoul); Jeong Ho Park (Seoul National University Hospital, Seoul); Kyung Won Lee (Keimyung University Dongsan Hospital, Daegu); Won Chul Cha (Samsung Medical Center, Seoul); Kyoung Jun Song (Boramae Medical Center; Seoul); Young Sun Ro (Seoul National University Hospital, Seoul); Kim Joo Yeong (Seoul National University Hospital, Seoul); Yu Jin Lee (Seoul National University Hospital, Seoul); Sungwoo Moon (Korea University Ansan Hospital, Gyeonggi)

**Singapore**

Michael YC Chia (Tan Tock Seng Hospital); Han Nee Gan (Changi General Hospital); Ling Tiah (Changi General Hospital); Benjamin SH Leong (National University Hospital); Wei Ming Ng (Ng Teng Fong General Hospital); Desmond R Mao (Khoo Teck Puat Hospital); Shir Lynn Lim (National University Heart Centre Singapore); Nausheen E Doctor (Sengkang General Hospital); Wei Ling Tay (Ng Teng Fong General Hospital); Shun Yee Low (Sengkang General Hospital); Lai Peng Tham (KK Women’s and Children’s Hospital); Si Oon Cheah (Urgent Care Clinic International); E-Shaun Goh (Woodlands Health); Yih Yng Ng (National University of Singapore); Yue Yen Lee (Tan Tock Seng Hopital); Sarah EM Tan (Tan Tock Seng Hopital); Gayathri Nadarajan (Singapore General Hospital); Ivan SY Chua (Singapore General Hospital); Shalini Arulanandam (Ministry of Home Affairs); Colin K Tan (Singapore Armed Forces); Dehan Hong (Singapore Armed Forces); David Pflug (Singapore Civil Defence Force); Fahad Siddiqui (Duke-NUS Medical School); Liu Nan (Duke-NUS Medical School); Shu-Ling Chong (KK Women’s and Children’s Hospital)

**Taiwan**

Matthew Huei-Ming Ma (National Taiwan University Hospital Yunlin Branch, Douliou); Patrick Chow-In Ko (College of Medicine, National Taiwan University, Taipei); Chih-Hao Lin (National Cheng Kung University, Tainan); Chan-Wei Kuo (Chang-Gung Memorial Hospital, Taoyuan); Wen-Chu Chiang (National Taiwan University Hospital, Taipei)

**Thailand**

Jirapong Supasaowapak (Rajavithi Hospital, Bangkok); Nathida Sumetchotimaytha (Rajavithi Hospital, Bangkok); Nalinas Khunklai (Rajavithi Hospital, Bangkok); Pairoj Khruekarnchana (Rajavithi Hospital, Bangkok); Sattha Riyapan (Siriraj Hospital, Bangkok), Borwon Witt (Maharaj Nakorn Chiang Mai Hospital, Chiang Mai); Chanodom Piankusol (Maharaj Nakorn Chiang Mai Hospital, Chiang Mai); Thammapad Piyasuwankul (Prince of Songkla University, Songkla)

**Malaysia**

Sarah Abdul Karim (Hospital Sungai Buloh, Selangor); Kwanhathai Darin Wong (Hospital Pulau Pinang, Penang); Nik Hisamuddin Nik AB Rahman (University Sains Malaysia, Kelantan); Boon Yang Chia (Sarawak General Hospital, Sarawak); Aik Howe Teo (Hospital Pulau Pinang, Penang); Mook Yuang Low (Miri General Hospital, Sarawak); Sheue Fen Ong (Hospital Sultanah Bahiyah, Kedah)

**India**

Govindaraju Venkata Ramana Rao (EMRI Green Health Services, Telangana); Rajanarsing HV Rao (EMRI Green Health Services, Telangana); Vimal M (EMRI Green Health Services, Telangana); TV Ramakrishnan (Sri Ramachandra Medical College and Research Institute, Chennai)

**Pakistan**

Munawar Khursheed (National Institute of Cardiovascular Diseases, Karachi); Nadeem Ullah Khan (Aga Khan University, Karachi); Uzma Khan (Aga Khan University, Karachi); Junaid Abdul Razzak (Aga Khan University, Karachi); Fareed Hassaan (Aga Khan University, Karachi); Noor Baig (Aga Khan University, Karachi)

**Philippines**

Faith JC Mesa-Gaerlan (Southern Philippines Medical Center, Davao); Patrick J Tiglao (Corazon Locsin Montelibano Memorial Regional Hospital, Bacolod); Bernadett P Velasco (East Avenue Medical Center, Manila); Pauline Convocar (Corazon Locsin Montelibano Memorial Regional Hospital, Bacolod); Kenneth Doya Nonesa (Southern Philippines Medical Center, Davao); April B. Llaneta (University of the Philippines, Manila); Marrie Vyne C Shakya (East Avenue Medical Center, Manila); Nerissa Sabarre (Pasig City General Hospital, Manila); Richard Santos (Pasig City General Hospital, Manila)

**China**

Wenwei Cai (Zhejiang Provincial People's Hospital, Zhejiang); Ander SA Zhou (Zhejiang Provincial People's Hospital, Zhejiang); Shao Fei (Beijing Chaoyang Hospital, Beijing)

**Vietnam**

Do Ngoc Son (Bach Mai Hospital, Hanoi); Dat Anh Nguyen (Bach Mai Hospital, Hanoi); Trong Ai Quoc Hoang (Hue Central General Hospital, Hue); Ton Thanh Tra (Cho Ray Hospital, Ho Chi Minh) Pham Dinh Quyet (115 Emergency Center, Ho Chi Minh); Chinh Quoc Luong (Bach Mai Hospital, Hanoi); Dai Quoc Khuong (Bach Mai Hospital, Hanoi, Vietnam); Tuan Anh Nguyen (Bach Mai Hospital, Hanoi); Thang Xuan Vu (Bach Mai Hospital, Hanoi); Thanh Nguyen (Hanoi 115 Emergency Center, Hanoi); Dat Tuan Nguyen (Bach Mai Hospital, Hanoi); Huan Huu Nguyen (Agriculture General Hospital, Hanoi); Hung Quang To (Vinh Phuc Provincial General Hospital, Vinh Phuc); Hai Minh Truong (Vietnam-Czechoslovakia Friendship Hospital, Hai Phong); Long Hoang Le (115 Emergency Center, Ho Chi Minh); Hung Trong Nguyen (115 Emergency Center, Ho Chi Minh); Trang Thuy Nguyen (115 Emergency Center, Ho Chi Minh)

**United Arab Emirates**

Ghulam Yasin Naroo (Rashid Hospital, Dubai); Omer Ahmed Alsakaf (Dubai Corporation for Ambulance Services, Dubai); Yadgir Tanveer Ahmed Mohd Ishaque (Dubai Corporation for Ambulance Services, Dubai); Assim Alhumodi (Abu Dhabi Police GHQ, Abu Dhabi); Muayyad Ibrahim Abumallouh (Dubai Corporation for Ambulance Services, Dubai)

**Lebanon**

Mazen El Sayed (American University of Beirut Medical Center, Beirut)
